# High road mortality during female-biased larval dispersal in an iconic beetle

**DOI:** 10.1007/s00265-020-02962-6

**Published:** 2021-01-16

**Authors:** Topi K. Lehtonen, Natarsha L. Babic, Timo Piepponen, Otso Valkeeniemi, Anna-Maria Borshagovski, Arja Kaitala

**Affiliations:** 1 Department of Ecology and Genetics, University of Oulu, Post Box 3000, 90014 Oulu, Finland; 2 Tvärminne Zoological Station, University of Helsinki, J.A. Palménin tie 260, 10900 Hanko, Finland; 3 Organismal and Evolutionary Biology, University of Helsinki, PO Box 65, 00014 Helsinki, Finland; 4 School of Biological Sciences, Monash University, Clayton Campus, Melbourne, Victoria 3800 Australia

**Keywords:** Dispersal, Ecological trap, Habitat, Life history, Mortality, Urbanisation

## Abstract

**Abstract:**

Animals often disperse from one habitat to another to access mates or suitable breeding sites. The costs and benefits of such movements depend, in part, on the dispersing individuals’ phenotypes, including their sex and age. Here we investigated dispersal and road-related mortality in larvae of a bioluminescent beetle, the European common glow-worm, *Lampyris noctiluca*, in relation to habitat, sex and proximity of pupation. We expected these variables to be relevant to larval dispersal because adult females are wingless, whereas adult males fly when searching for glowing females. We found that dispersing glow-worm larvae were almost exclusively females and close to pupation. The larvae were often found on a road, where they were able to move at relatively high speeds, with a tendency to uphill orientation. However, each passing vehicle caused a high mortality risk, and we found large numbers of larvae run over by cars, especially close to covered, forest-like habitat patches. In contrast, adult females in the same area were most often found glowing in more open rocky and grassy habitats. These findings demonstrate an underappreciated ecological strategy, sex-biased dispersal at larval phase, motivated by different habitat needs of larvae and wingless adult females. The results are also consistent with roads being an ecological trap, facilitating dispersal and presumably females’ signal visibility but causing severe larval mortality just before the reproductive stage. Hence, in addition to the previously recognised threats of urbanisation, even low traffic volumes have a high potential to negatively affect especially females of this iconic beetle.

**Significance statement:**

Animals sometimes need to move from one habitat to another to find mating partners or breeding sites. We found this need to result in strongly female-biased larval dispersal in the European common glow-worm, a beetle known for the glow of wingless females that attract flying males to mate. Female larvae moving between habitats often used a road or trail but perished in high numbers when run over by cars. Hence, roads are likely to be ecological traps for the female glow-worm larvae, attracting them during dispersal, but causing grave mortality. The sex-biased larval dispersal, demonstrated in this study, is a poorly known ecological strategy that was found to be very risky in a human-modified landscape.

**Supplementary Information:**

The online version contains supplementary material available at 10.1007/s00265-020-02962-6.

## Introduction

Most environments are heterogeneous both in space and time, with dispersal, a key process in population dynamics and evolutionary ecology, being a common response to this heterogeneity (Johnson and Gaines 1990; Roff and Fairbairn 2007). An individual moving to a new location or environment can benefit from access to a desired habitat or breeding ground (Verhulst et al. 1997; Schjørring 2002; Lin and Batzli 2004) or from avoidance of pathogens, predators, resource competition, sexual competition or inbreeding (Perrin and Mazalov 2000; Yoder et al. 2004; Matthysen 2005; Nagy et al. 2013; Alcalay et al. 2018). Such benefits aside, movement between environments may increase mortality and entail other significant costs (Van Vuren and Armitage 1994; Baker and Rao 2004; Bonte et al. 2012), including those from an increased predation risk (Larsen and Boutin 1994; Yoder et al. 2004; Rittenhouse et al. 2009), energy expenditure (Stobutzki 1997; Baker and Rao 2004) and straying into unsuitable habitat or away from potential mates (Burgess et al. 2012). Hence, whether an individual should engage in such movements depends on the balance between the associated costs and benefits (Johnson and Gaines 1990; Bonte et al. 2012).

The costs and benefits of dispersal are affected not only by the environment but also the individual’s own phenotype (O’Riain et al. 1996; Bowler and Benton 2005; Clobert et al. 2009), including its body size (Legagneux et al. 2009; Cote and Clobert 2010) and age (Altwegg et al. 2000). The costs and benefits of dispersal may also differ between the sexes, for example, due to sex differences in resource competition, inbreeding avoidance and competition for mates (Dobson 2013). In the stag beetle, *Lucanus cervus*, for example, males fly at higher altitudes, whereas females more likely stay on the ground or fly at lower heights while searching for suitable oviposition sites, resulting in higher female mortality by vehicle collisions (Harvey et al. 2011). Such differences in fitness consequences may also result in sex differences in dispersal strategies (Perrin and Mazalov 2000; Gros et al. 2008). Indeed, sex differences in dispersal have been documented in reptiles (Olsson and Shine 2003), amphibians (Austin et al. 2003), fish (Hutchings and Gerber 2002; Bekkevold et al. 2004; van Dongen et al. 2014) and invertebrates (Caudill 2003; Sundström et al. 2003; Beirinckx et al. 2006). Considering insects, one potentially important driver of sex biases in dispersal is female flightlessness. Flightlessness allows higher resource allocation to egg production, while retained flight ability in males increases the probability of finding a mate (Roff 1990; South et al. 2010).

Regarding environmental factors, urbanisation and other anthropomorphic changes have resulted in habitat fragmentation and interfered with movements of many animals (Fahrig 2007; Bonte et al. 2012). Light pollution, for example, is known to mislead animals from their evolved baseline movement patterns (Degen et al. 2016; Owens et al. 2020), which may also reduce mate detection, as in Lampyrid beetles (Firebaugh and Haynes 2016; Lewis et al. 2020; Owens et al. 2020). Roads have been recognised as another major human-induced dispersal hazard especially for many birds and mammals (Lodé 2000; Ramp et al. 2006; Benítez-López et al. 2010; Kociolek et al. 2011). While data on road-associated mortality in small animals are sparser, there are nevertheless observations that smaller animals, such as reptiles (Rosen and Lowe 1994; Markle et al. 2017), amphibians (Fahrig et al. 1995; Hels and Buchwald 2001) and invertebrates (Rao and Girish 2007; Muñoz et al. 2015; Keilsohn et al. 2018), can also be negatively affected, especially during their reproductive season and other activity peaks (Beaudry et al. 2010; Harvey et al. 2011). Nevertheless, road ecology research in insects has been particularly scant (Baxter-Gilbert et al. 2015; Muñoz et al. 2015), despite roads potentially affecting insect abundance and diversity negatively (Muñoz et al. 2015; Keilsohn et al. 2018) and presumably killing billions of pollinating insects each year (Baxter-Gilbert et al. 2015).

In this study, we investigated dispersal and road-related mortality in larvae of the European common glow-worm, *Lampyris noctiluca* L. (Coleoptera: Lampyridae) in relation to their age, sex and habitat. In fireflies, such as the glow-worm, female neoteny and flightlessness are linked to sexual size dimorphism and increased female fecundity while increasing the need for female sexual signalling (South et al. 2010). In particular, wingless and sedentary glow-worm females need to find a visible spot where they then emit a greenish glow during the night to attract flying males to mate. There is considerable variation between females in their mate attraction success (Hickmott and Tyler 2011; Hopkins et al. 2015; Lehtonen and Kaitala 2020), and, because adult glow-worms do not eat (i.e. are capital breeders), any delays in mating are costly (Wing 1989; Hopkins 2018). At most, adults can survive for only a few weeks and after mating the female lays her eggs and then dies within hours (Dreisig 1971; Tyler 2002; personal observations). During the larval phase (from egg to imago) of 3 years (Tyler 2002), glow-worms eat predominately snails and occasionally slugs and earthworms (Tyler 2002; personal observations). In larvae, nocturnal glow signals are thought to advertise their unpalatability to would-be predators (Underwood et al. 1997; De Cock and Matthysen 2001, 2003; Tyler et al. 2008). Interestingly, some observations suggest that significant numbers of last instar larvae are on the move during the daytime prior to the mating season, which may contribute to dispersal within and between habitats, despite adult males being able to fly (Tyler 2002; Atkins et al. 2017). It is plausible that such timing of dispersal could allow the larvae to shift from the relatively lush larval hunting grounds to more open sites favoured by adults, especially displaying females.

The current study had multiple objectives related to life-history-specific movements, and the risk roads may pose during such dispersal. First, we investigated whether the larvae that are found moving along open habitats (especially roads and roadsides) during the daytime will pupate soon rather than being in earlier stages of their multi-year life cycle. We expected to observe the former, if the main purpose of these larval movements is the hypothesised habitat shift. Second, we assessed the sex ratio of the dispersing larvae. We hypothesised that because adult males can fly but females do not, and hence, only adult females need immediate access to open displaying grounds, most dispersing larvae would be females. Third, because of the observations of dispersing larvae in open habitats (such as trails and roads), we hypothesised that roads can be a major threat to them. As detailed below, two different approaches were used to assess their road mortality. Fourth, we measured the speed and orientation of the dispersing larvae to better understand their dispersal potential and its driving factors. Finally, we investigated habitat use of the dispersing larvae and adult females. This was done to further assess the hypothesis that the species has adopted the ecological strategy of larval dispersal from covered habitats to the sites of female sexual glow display (more open habitats).

## Materials and methods

The study was conducted in the vicinity of Tvärminne Zoological Station, southern Finland (59° 50.7′ N; 23° 14.9′ E) in 2019 and 2020. The study site also included a paved country road (J.A. Palménintie), which is ~ 4.7-m wide and runs for roughly 2.5 km westwards from the research station before connecting to a larger road. Aside from buildings belonging to the research station at the end of the road, there is a low number (< 10) of houses along the road, and only a short stretch of it has streetlights (~ 100 m, on one side). The study site was selected due to the logistic convenience of it being close to the research station and because previous studies had showed that glow-worms inhabit the area (Borshagovski et al. 2019, 2020; Elgert et al. 2020, 2021; Lehtonen and Kaitala 2020). More generally, the European glow-worm has a wide range from Iran (N32°) to central Finland (N64°) (Samin et al. 2018; Borshagovski et al. 2020), with the species being on the decline across Europe, as are many other Lampyrid species globally (Lewis et al. 2020).

### Age and sex ratio of dispersing larvae

To assess the sex ratio of dispersing larvae and to investigate whether they are close to the pupation stage, we collected the larvae that were found moving in open areas, either along the country road (see above) or elsewhere in the vicinity of the research station. The larvae were collected between May 20 and June 3 in 2019 (*N* = 32 collected from the study area, 6 from elsewhere in Finland on May 15–17) and May 23 and June 4 in 2020 (*N* = 30 collected of which 26 were assessed). The collection periods coincided with the phase when glow-worm larvae were observed to be particularly active, i.e. after the weather had sufficiently warmed up in May but before significant numbers of larvae had presumably settled for pupation in June (personal observations). In 2019, after the larvae had been brought to the research station, they were placed in arenas of 100 cm × 20 cm × 20 cm (length × width × height), each of which was covered by a fine plastic mesh to prevent the larvae from escaping. The arenas were placed in a non-insulated shed with a glass roof and were therefore exposed to natural light and temperature cycles. Each arena contained a 2-cm layer of soil on the bottom, two bivalve shells as shelters and two Helicidae snails as a food source. Snails that were eaten by the larvae were replaced with new ones. Two larvae were placed in each arena. The arenas and the larvae in them were checked daily for a month or until they pupated. If a larva pupates, its sex is easy to determine from the presence of wing buds in a male, but not female, pupa. We successfully investigated 27 larvae. In addition, 2 died and 9 escaped due to the mesh failing to cover the arena properly in these cases. Therefore, in 2020, larvae were kept individually in cylinder-shaped plastic containers that were 8 cm in diameter and 7 cm in height, each having a firm lid with a fine mesh section for air exchange. Each container also had moisturised leaves, moss and one snail (as a food source, replaced if eaten). The containers were placed in another shed where the larvae were exposed to subdued natural light (through a window) and temperatures similar to, but in the afternoons slightly lower than, outside. In 2020, we assessed pupation of 26 larvae. Of those, we additionally recorded how many had eaten at least one snail. After the experiment, the individuals were released back to the wild close to the original site of collection.

### Distribution of road-killed larvae

**Fig. 1:**
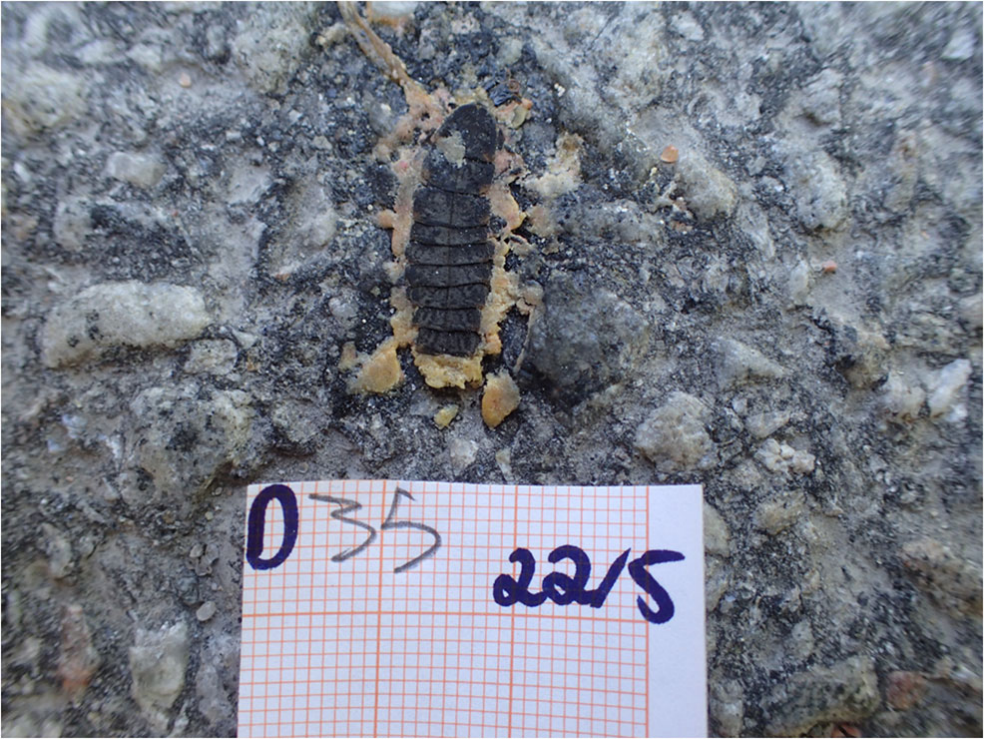
Vehicle-killed common glow-worm larva close to Tvärminne Zoological Station, Finland. The image includes an identification code and 1-mm grid paper as a scale

Given that larvae can be found in open landscapes, such as roadsides, in 2019 we assessed the numbers of larvae that were killed by cars (and potentially other road users) and determined the habitat types parallel to the kill locations. For this purpose, we counted and photographed all larvae that were found dead (presumably run over by a vehicle, see Fig. 1) on the 2.5-km stretch of the road within the study site. Hence, we used the road as a form of transect for assessing larval distribution (see Taylor and Goldingay 2004; Harvey et al. 2011). This approach has also shown to be a useful indicator of the numbers of individuals, for instance, in the stag beetle (Harvey et al. 2011). The assessment was conducted over three consecutive days (afternoons and evenings), from May 21 to May 23 in 2019. Two researchers walked in the same direction on opposite sides of the road. This helped to ensure that most larvae were detected and identified, based on the species-specific segmentation, colour markings and colourful innards (Fig. 1). When a road-kill larva was spotted, we marked the GPS coordinates of the location using the “drop pin” function in the Google Maps application (Google LLC, Android and iOS versions), independently operated by two smart phones (the second one being used as a backup). We then photographed the specimen (using an Olympus Tough TG-4 digital camera) next to a 1-mm grid paper scale and identification tag (Fig. 1). Next, we recorded the habitat type on the side of the road that the measured specimen was closer to. Typically, the habitat type was the same on both sides. We used the following four categories that effectively captured the main habitat types occurring along the road: (1) rocky formations (predominantly bedrock), (2) open grassland and fields, (3) understory with shrubs and bushes and (4) forest (defined as the dense occurrence of mature trees). The commonness of these habitat types differed, with the more covered habitat types (3 and 4) being more common in the study area than the open ones (1 and 2). After finishing the first day of survey, the next day we continued from exactly the same location where the previous session had been finished. The above measures—two people cross-checking between each other, photographs of each individual, identification tags, GPS coordinates and keeping close track of the starting and finishing locations—ensured that no specimen was measured more than once. The traffic volume was estimated as 5–15 vehicles/h during mornings and afternoons and 0–10 vehicles/h during evenings and nights at the time of the study in 2019.

### Distribution of displaying adult females

To compare the numbers and habitats of dispersing larvae (as described above) to those of adult females, we sampled the same road during 20 nights between June 12 and July 3 in 2019, a period that covered the breeding season from the start to beyond its peak. In particular, 1 or 2 investigators walked from one end of the road to the other and then back during the peak period of the night when glow-worm females were expected to be displaying (at ~ 0:15–1:45). The exact starting time and the starting point along the road were varied haphazardly between the nights so that the time of the night a certain section of the road was assessed varied between the nights. When two investigators participated, they started at the opposite ends of the road. Upon detecting a glowing female from the road, we recorded, as above, the GPS coordinates of the spot using the “drop pin” function on Google Maps. The female was then collected and used in research unrelated to this study. The habitat type of the location was later recorded using the same four categories as above. The investigator who assigned the habitat types was the same for the assessments of both adult females and larvae, but kept blind to the results of the assay of adult females.

### Larval movement and road mortality rate

To assess the speed and direction of glow-worm larvae’s movements and to get an estimate of their mortality on the road per car, in 2020 (on May 20–June 4, at varying hours between 09:00–21:30) we patrolled the country road and elsewhere in the study site. Once a larva was detected, it was followed, and its movements were tracked using markings made with a coloured chalk or, when this was not practical, using small, coloured pebbles. In addition, a different marking colour was used every 5 min to record the location of the larva at that point of time. This allowed us to measure the length of the track the larva had walked in 5 min. Each larva (*N* = 33) was followed for 5–55 min (mean: 21 min). When more than one 5-min value was available for a larva, we used the average over these values as an estimate of its moving speed. When the larva was walking on the road while at least one car passed, we also recorded the number of the vehicles (with the researcher sidestepping to a randomised side of the road) and whether the larva survived. If the larva was killed, the replicate was ended. If the larva survived, did not escape and we had an empty collection box available, it was collected and used in the experiment “Age and sex ratio of dispersing larvae” (see above). Due to COVID-19 movement restrictions at the time (late May–early June in 2020; see Moisio 2020), the traffic volume on the road was lower than usual (average, 3 cars/h; range, 0–12; *N* = 23 assessments of 15–60 min).

## Results

### Age and sex ratio of dispersing larvae

In 2019, all 27 larvae pupated, with pupation observed 21.9 ± 1.1 (mean ± SE) days from the date of collection (range, 11–34 days, including multiple days of inactivity preceding pupation). Of these, 26 were females and 1 was male, implying a highly female-biased sex ratio (one-tailed binomial distribution test, *P* < 0.0001). Similarly, in 2020, 25 of the 26 larvae started pupation within a month (pupation time, 21.4 ± 0.5 days; range, 16–26 days), and, of these, 23 were females and 2 were males (one-tailed binomial test, *P* < 0.0001). In 2020, when we also monitored feeding of the larvae after they had been collected, only 7 out of 26 fed on the easily accessible food source (significantly fewer ate than did not eat: one-tailed binomial test, *P* = 0.015), suggesting that a majority of larvae were so close to pupation that they had already ceased eating.

### Distributions of road-killed larvae and displaying adult females

**Fig. 2:**
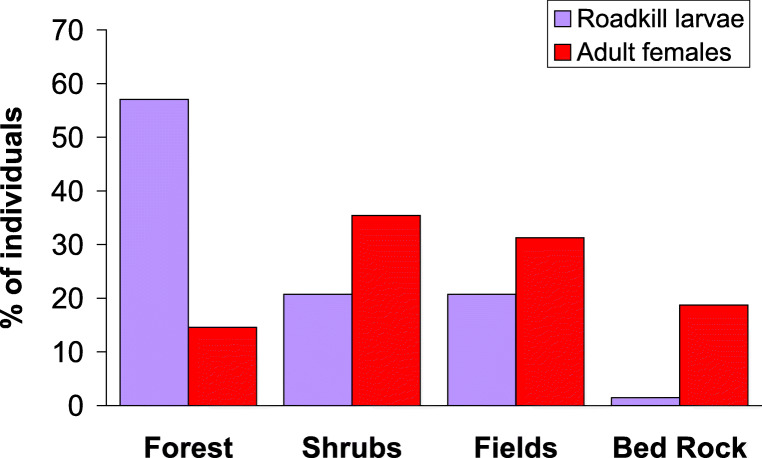
Habitat types next to which vehicle-killed glow-worm larvae were found and in which adult females were glowing later in the same season (2019, southern Finland). More open habitat types are placed further right in the figure

**Fig. 3:**
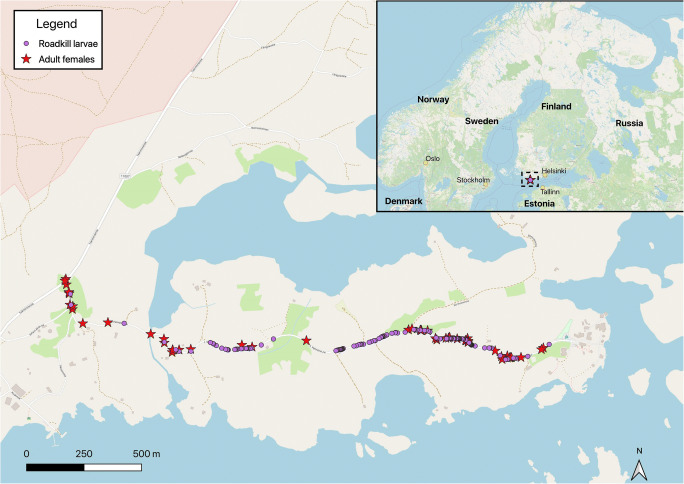
The occurrence of road-kill glow-worm larvae (*N* = 135) and adult females (*N* = 48) in 2019, at the study site in southern Finland

In 2019, we found 135 glow-worm larvae killed by vehicles along the 2.5-km road with low traffic volume. The adult female assay, in turn, resulted in detection and mapping of 48 displaying females. The habitats where the larvae and females were found differed (*G* test of independence, *G* = 35.53, df = 3, *P* < 0.0001). In particular, adult females were often found in relatively open habitats next to fields or on patches of bedrock whereas larvae were killed in the highest numbers next to the forest (Fig. 2). The two groups also differed in terms of the section of the road where they were found (500-m distance categories in relation to the end of the road: *G* test of independence, *G* = 20.78, df = 4, *P* = 0.0004; Fig. 3). The conclusion is the same when absolute distances are assessed.

### Larval movement and road mortality rate

Of the 33 larvae followed in 2020, 29 moved at least a part of the observation time on the paved road and 20 larvae moved at least a part of the time on a different substrate, such as a grassy roadside, unpaved trail or sandy substrate. Their walking speed on the paved road was 28 ± 2 cm/min and on all other substrate types combined 8 ± 1 cm/min. This implies a faster rate of movement on the paved road (paired *t* test for those individuals with both measures available: *t*_15_ = 8.765, *P* < 0.0001). In addition, 15 of the larvae were observed moving on a non-level terrain or part of the road. Of these, 3 were heading downhill, 9 uphill and 3 took both orientations during the observation period. This implies a marginally non-significant tendency for an uphill orientation to be more common than a downhill one (binomial distribution test, one-tailed binomial test, *P* = 0.074). Despite the particularly low traffic volumes in 2020, 17 of the observed larvae were on the road while at least one car passed, with 3 of the total of 28 encounters being fatal, giving an overall mortality of 11% per car.

## Discussion

Glow-worm larvae that were found moving on a road or in other open habitats were almost exclusively females, close to pupation. In addition, even a low volume of traffic on a country road resulted in high larval mortality (a spot sample yielded 135 dead larvae on a road of 2.5 km, whereas 48 adult females were found glowing in the area in nightly assays during 3 weeks of the peak breeding season). Indeed, when moving on the road, a larva was found to have roughly 10% probability of getting killed by each passing car (during a period when the traffic volume was low due to movement restrictions). The mortality rate is in line with the expected, given the width of standard car tracks (2 × 20 cm when driving straight ahead) and the < 5-m width of the road (with the outermost edges being rarely used). This finding indicates that many roads, especially those with moderate to high traffic volumes and running through glow-worm habitats, can be significant population sinks to these animals. Moreover, we found that larvae were killed on the road in the highest numbers next to the forest habitat, whereas adult glow-worm females were most often found associated with more open rocky, grass and field habitats. Furthermore, when there were vertical differences in the immediate terrain, three times more dispersing glow-worm larvae were heading uphill than downhill, albeit this difference was not statistically significant.

By dispersing from one habitat to another, females may increase their access to mates (Bowler and Benton 2005; Arlt and Pärt 2008). Here, particularly female larvae close to the pupation stage were found to disperse by moving from their more forested hunting grounds, presumably towards drier and more open habitats, where adult females were often found performing their nightly glow displays. The suitability of a given site for the displays of an adult female may be affected by at least behavioural interactions among females, population size (driving competitive interactions), visibility to ensure effective mate attraction and egg-laying opportunities nearby (Hopkins 2018; Borshagovski et al. 2019; Elgert et al. 2020, 2021; Lehtonen and Kaitala 2020). Additionally, a female’s egg-laying site should not be located too far from where the newly hatched offspring can find their first meal after hatching later in the season. In this respect, it is currently not known whether, after the larval phase, female glow-worms return to their natal site or habitat for reproduction, as seen in some species (Stamps and Swaisgood 2007; Dolný et al. 2013). Adult male glow-worms, in turn, can and do fly, which we suggest to be the main reason for why most dispersing larvae were found to be females. Despite the flight capability of males, our results support the idea that the larval phase also contributes considerably to dispersal in the species (for another beetle species, see Traugott 2002). Larvae were also found to walk at notable speeds, which make them capable of reaching adjacent habitats within a sensible time.

Aside from the apparent need to shift habitats between the larval and adult stages, we consider the following four factors that may also have contributed to the observed differences in habitat use. First, it is possible that detection of adult females was more likely in open, rather than covered, habitats due to potential differences in visibility from the roadside. However, we consider it unlikely that this could have a large influence on our results, because the distance from the road at which we detected females was relatively similar between the habitats (~ 4–5 m). We assume that the low variability in our detection distances was due to grass and irregular rock surfaces blocking the horizontal visibility to an investigator walking on the roadside roughly as much as bushes or trees did, although for flying males the more covered habitats presumably entail greater visibility and manoeuvring barriers. Second, a high larval mortality in a specific habitat may result in fewer females surviving to adulthood in that habitat. This mechanism could contribute to the observed differences in habitat use, especially if larvae are attracted to roads more commonly next to covered (forest) habitats. Third, any number of adult females detected during this study may have arrived from locations away from the road. If the habitat types differ with regard to the distance from which females arrive to their glowing spots along the road, the observed female numbers might bias towards that specific habitat. Fourth, mate attraction success of displaying females is known to be negatively affected by the proximity of rivals (Hopkins 2018; Lehtonen and Kaitala 2020), which may also be driving females to disperse farther away from each other before or after pupation (see Borshagovski et al. 2019), resulting in increased dispersal away from habitats with high larva densities (even in the absence of a specific target habitat type). More generally, our results are compatible with the idea that dispersal, even in females, can be driven by density-dependent factors, such as competition for breeding opportunities (Dobson and Jones 1985; Matthysen 2005; Arlt and Pärt 2008).

Roads have been recognised as a major anthropogenic disturbance for a range of taxa. Besides sublethal individual effects, such as chronic stress (Bourbonnais et al. 2013; Bhattacharjee et al. 2015), many animal populations are negatively affected by road mortality, resulting in decreased population sizes and densities (Fahrig et al. 1995), shifts in sex ratios (Gibbs and Steen 2005) and reduced genetic diversity (Noël et al. 2007; Jackson and Fahrig 2011). Road mortality can negatively affect abundance or diversity also in insects (Muñoz et al. 2015), potentially causing deaths of billions of pollinators each year (Baxter-Gilbert et al. 2015). Here, we found not only a high number of larvae killed by vehicles but also that, for a larva on the road, each vehicle posed a high mortality risk. Indeed, beetle larvae being killed by cars in such high numbers may seem surprising, given that the traffic volume on the study road was very low. Thus, a higher traffic volume is likely to be even deadlier for the dispersers (Muñoz et al. 2015; Keilsohn et al. 2018). However, a higher rate of traffic may also deter animals away from the road, and in some small animals, the association between traffic volume and the number of individuals being killed by vehicles has been bell-shaped or even negative (Eberhardt et al. 2013; Grilo et al. 2015). In this respect, traffic volumes in the study area were unusually low in May 2020, because of COVID-19-related movement restrictions and the research station being closed from the public. We also note that in 2019, the exact number of larvae we counted represents a spot sample of road-kills that have presumably been accumulating over several days during, or close to, the yearly peak activity of glow-worm larvae (personal observations). Rainfall effectively washes larva remains away from the road (personal observations), and significant rainfall had taken place approximately a week prior to the survey. In addition, some bodies of larvae are likely to disappear from the road continuously (and hence before being counted, see Santos et al. 2011; Guinard et al. 2012), albeit larvae of Lampyrids are thought to be relatively unpalatable to many potential predators and scavengers (Underwood et al. 1997; De Cock and Matthysen 2001, 2003; Tyler et al. 2008).

One potential reason for the observations of larvae on roads late in the spring is the apparent suitability of that type of terrain both for dispersal (high larval movement rates found in this study) and as display sites for adult females. Hence, roads may act as ecological (or evolutionary) traps in which habitat quality cues are disassociated with the actual fitness consequences of moving along the structure (Kokko and López-Sepulcre 2006; Hale and Swearer 2015). For example, dragonflies and mayflies patrol paved roads instead of the natural habitat of still bodies of water, with the two habitats offering similar habitat cues (Horváth et al. 1998; Kriska et al. 1998). Indeed, the movement choices of many animals may not be optimal or well adapted in human-modified landscapes (Fahrig 2007). Our results also show that in glow-worms, mortality resulting from the propensity to disperse on a road is highly female-biased. Sex-biased road mortality due to behavioural differences has also been found, for example, in adult stag beetles (female bias, Harvey et al. 2011) and green iguanas, *Iguana iguana* (male bias, Rodda 1990).

Regarding such human-induced environmental changes, light pollution has been suggested as an important disrupter of behaviour, contributing to population declines in nocturnal species, such as glow-worms and other fireflies (Lewis et al. 2020). In particular, the ability of flying males to find glowing females is compromised under artificial light (Bird and Parker 2014; Firebaugh and Haynes 2016; Elgert et al. 2020, 2021). Our results suggest that the high level of (female-biased) road mortality only weeks before pupation constitutes another, at least locally severe, hazard to glow-worms and other similarly behaving species, and it may therefore have contributed to their decline (see Lewis et al. 2020). Hence, mitigation of the negative road effects at least close to key habitats would probably be beneficial to the local populations. Potential mitigation measures could include small barriers that prevent larvae crawling to the road, increasing of the distance between the habitat edge and the road (Keilsohn et al. 2018), and avoidance of road use during the relatively short peak of larval dispersal.

To conclude, this study demonstrates how female glow-worms move between habitats towards the end of their larval stage, shortly before pupation. The results suggest that larval dispersal is an often overlooked ecological strategy that is likely to be driven by different habitat needs of the larvae and adults. Moreover, the road surface seems to function as an ecological trap, with the larval movements using that landscape feature resulting in a high, female-biased mortality. These findings show how urbanisation, even in the form of a country road with low traffic volumes, can affect dispersal and survival of glow-worms, and potentially other small animals, on their way to reproduce.

## Supplementary information

ESM 1 (XLS 95 kb)

## Data Availability

Data are available as supplementary material.
